# Direction-selective modulation of visual motion rivalry by collocated tactile motion

**DOI:** 10.3758/s13414-022-02453-y

**Published:** 2022-02-22

**Authors:** Gwenisha J. Liaw, Sujin Kim, David Alais

**Affiliations:** The University of Sydney, School of Psychology, Sydney, Australia

**Keywords:** Binocular rivalry, Visuo-tactile motion perception, Multisensory integration

## Abstract

Early models of multisensory integration posited that cross-modal signals only converged in higher-order association cortices and that vision automatically dominates. However, recent studies have challenged this view. In this study, the significance of the alignment of motion axes and spatial alignment across visual and tactile stimuli, as well as the effect of hand visibility on visuo-tactile interactions were examined. Using binocular rivalry, opposed motions were presented to each eye and participants were required to track the perceived visual direction. A tactile motion that was either a leftward or rightward sweep across the fingerpad was intermittently presented. Results showed that tactile effects on visual percepts were dependent on the alignment of motion axes: rivalry between up/down visual motions was not modulated at all by left/right tactile motion. On the other hand, visual percepts could be altered by tactile motion signals when both modalities shared a common axis of motion: a tactile stimulus could maintain the dominance duration of a congruent visual stimulus and shorten its suppression period. The effects were also conditional on the spatial alignment of the visual and tactile stimuli, being eliminated when the tactile device was displaced 15 cm away to the right of the visual stimulus. In contrast, visibility of the hand touching the tactile stimulus facilitated congruent switches relative to a visual-only baseline but did not present a significant advantage overall. In sum, these results show a low-level sensory interaction that is conditional on visual and tactile stimuli sharing a common motion axis and location in space.

**Supplementary Information:**

The online version contains supplementary material available at 10.3758/s13414-022-02453-y.

## Introduction

In order to perceive our surroundings in a robust and coherent manner, the brain must integrate sensory information within and between modalities (Alais, Newell, & Mamassian, 2010a; Ernst & Bülthoff, 2004). Integration across the senses can play an important role in resolving perceptual ambiguities in vision, as shown by a number of recent studies (Alais, van Boxtel, et al., 2010b; Blake et al., 2004; Conrad et al., 2012; Lunghi et al., 2014; Lunghi & Alais, 2013, 2015; van Ee et al., 2009). However, the question of where sensory signals are combined along processing pathways is a matter of debate. Early models held that multisensory integration occurred beyond primary sensory cortices in multisensory association areas. For example, the human analogue of the ventral intraparietal cortex (hVIP) has been found to encode for both visual and tactile information with spatially aligned maps across modalities (Duhamel et al., 1998; Sereno & Huang, 2006). On the other hand, recent evidence suggests that multisensory interactions may instead be a feature of all cortical areas (Ghazanfar & Schroeder, 2006; Konkle & Moore, 2009). Transcranial-magnetic stimulation (TMS) applied over area MT, an early visual motion processing locus along the dorsal pathway, during tactile motion trials significantly impaired accuracy, reaction times and speed perception (Basso et al., 2012; Matteau et al., 2010; Ptito et al., 2009; Ricciardi et al., 2011). In addition, area MT has been found to respond to tactile motion discrimination in blind participants, which suggests that MT is also involved in tactile motion processing and is not exclusive for visual motion perception as traditionally thought (Amemiya et al., 2017; Moutoussis & Zeki, 2008).

Analogous effects across vision and touch have previously been reported for dynamic signals, highlighting similarities in visuo-tactile motion processing (Carter et al., 2008; Gori et al., 2011). For example, both vision and touch share comparable organisational principles when extracting and encoding motion signals over space and time (Pack & Bensmaïa, 2015; Pei et al., 2008). Behaviourally, visuo-tactile interactions have been demonstrated using adaptation phenomena such as motion aftereffects (MAE). Konkle and colleagues  (2009) found that prolonged exposure to visual motion in a given direction subsequently elicited the illusion of motion in the opposite direction in the tactile domain. Crucially, this MAE was replicated when the modalities were reversed. This finding is significant as adaptation phenomena show selectivity for spatial location and direction, which hints at a low-level sensory adaptation in the absence of awareness. This implies that vision and touch might rely on common neural substrates located earlier along processing pathways than previously assumed. One possible neural site is that of area MT, as it is specialised for motion perception and exhibits direction selectivity. The finding that tactile motion can elicit a visual MAE accord with studies that have observed MT activation for tactile signals in the absence of concurrent visual stimulation, thus supporting the idea of an early and supramodal representation of motion information (Born & Bradley, 2005; Kohn & Movshon, 2004; Tootell et al., 1995).

One hallmark feature of area MT is that its cells are direction-selective – when a grating was moved through the receptive field of a macaque MT neuron, it responded only to a narrow range of directions predominantly orthogonal to the grating’s orientation (Albright, 1984; Albright et al., 1984). This direction specificity has also been demonstrated in human MT using the bistable visual apparent-motion quartet, which consists of a pair of dots flashing in alternation at the two diagonals of an invisible square, resulting in participants switching between perceiving horizontal and vertical motion. Area MT was observed to show distinct activations for motion along the horizontal and vertical axes, with activation corresponding to the direction of motion perceived by the observer (Schneider et al., 2019). Given that the apparent-motion quartet has been replicated in the somatosensory system using vibro-tactile stimuli, the results point to a direction-selective component underlying tactile motion processing that is similar to vision (Carter et al., 2008). Importantly, a concurrently presented task-irrelevant visual motion quartet was found to increase dominance durations for a task-relevant tactile motion quartet only when both stimuli were directionally (i.e, moving in the same direction within the same axis) and spatially congruent (Conrad et al., 2012). These findings highlight spatial congruency as another factor for multisensory interactions, which is in accord with the spatial rule of multisensory integration. Indeed, it was previously observed that placing a haptic grating 30 cm away from the visual stimulus did not result in any cross-modal interactions, and size discrimination precision decreases as the spatial distance between collocated visuo-tactile stimuli increases (Gepshtein et al., 2005; Lunghi & Morrone, 2013). These results not only support the notion that motion processing across vision and touch are closely interlinked, they highlight direction and spatial alignment as integral factors in determining visuo-tactile interactions.

Another form of bistable percepts that has been used to study the processes underlying visuo-tactile interactions is binocular rivalry (Lunghi et al., 2010; Lunghi & Alais, 2013; Lunghi & Morrone, 2013). Binocular rivalry has proven to be a powerful method for probing the mechanisms behind visual consciousness as it elicits changes in visual awareness in the presence of physically constant stimuli (Alais, 2012; Alais & Blake, 2015). Moreover, binocular rivalry differs from other forms of bistable phenomena as the suppressed image is generally thought to be less susceptible to top-down modulations in the form of attention and working memory, which renders it a useful paradigm to examine low-level sensory interactions (Meng & Tong, 2004; Scocchia et al., 2014). Lunghi and colleagues previously used oriented haptic gratings to demonstrate that tactile input congruent with one of the rivalling gratings could influence rivalry dynamics when the haptic and visual stimuli were congruent in orientation, tightly matched in spatial frequency and spatially aligned (Lunghi & Alais, 2015; van der Groen et al., 2013). A similar effect has been observed for translational motion signals: congruent tactile motion promoted the dominance duration of the matching visual stimulus and shortened its periods of suppression, supporting an early interaction across modalities (Hense et al., 2019).

This study thus aims to extend previous findings using binocular rivalry to characterise the locus and nature of visuo-tactile interactions. As motion signals can serve as salient cues, the extent to which direction-congruent tactile signals are integrated in the presence and absence of visual awareness remains to be seen. We thus contrasted rivalry dynamics when visual and tactile stimuli moved along parallel versus orthogonal axes to investigate if a preferred axis of motion is shared across modalities. We also examined if visuo-tactile interactions would still occur when tactile motion was presented to spatially misaligned visual stimuli. Furthermore, most visuo-tactile studies that have utilised the rivalry paradigm have so far done so without visual feedback – participants were unable to see their stimulated hand (Hense et al., 2019; Lunghi & Alais, 2013; Lunghi & Morrone, 2013). Given that vision and somatosensation are closely linked to proprioception, it remains to be seen if hand visibility could facilitate visuo-tactile interactions by serving as an ecological cue within peripersonal space. The effect of hand visibility on rivalry dynamics was therefore also tested. Using a within-subjects approach, our results extend existing knowledge on visuo-tactile interactions to show that it is dependent on the alignment of motion axes and location in space across modalities.

## Materials and Methods

### Participants

A total of 14 participants (including the authors) with an average age of 27.1 ± 10 years (4 males) completed this experiment. All had self-reported normal or corrected-to-normal vision, and normal stereoacuity (as assessed by the Fly Stereo Acuity test; Vision Assessment Corporation, Elk Grove Village, IL, USA). All were right-handed, had no tactile impairments, and were naïve to the purposes of the experiment except for the authors. The data for three additional participants were excluded for not completing the experiment and for difficulties in achieving stable binocular fusion using a mirror stereoscope. This research was approved by the University of Sydney Human Research Ethics Committee. All participants gave written, informed consent before commencing the experiment and received $20 per hour for their participation.

### Apparatus and Stimuli

**Fig. 1:**
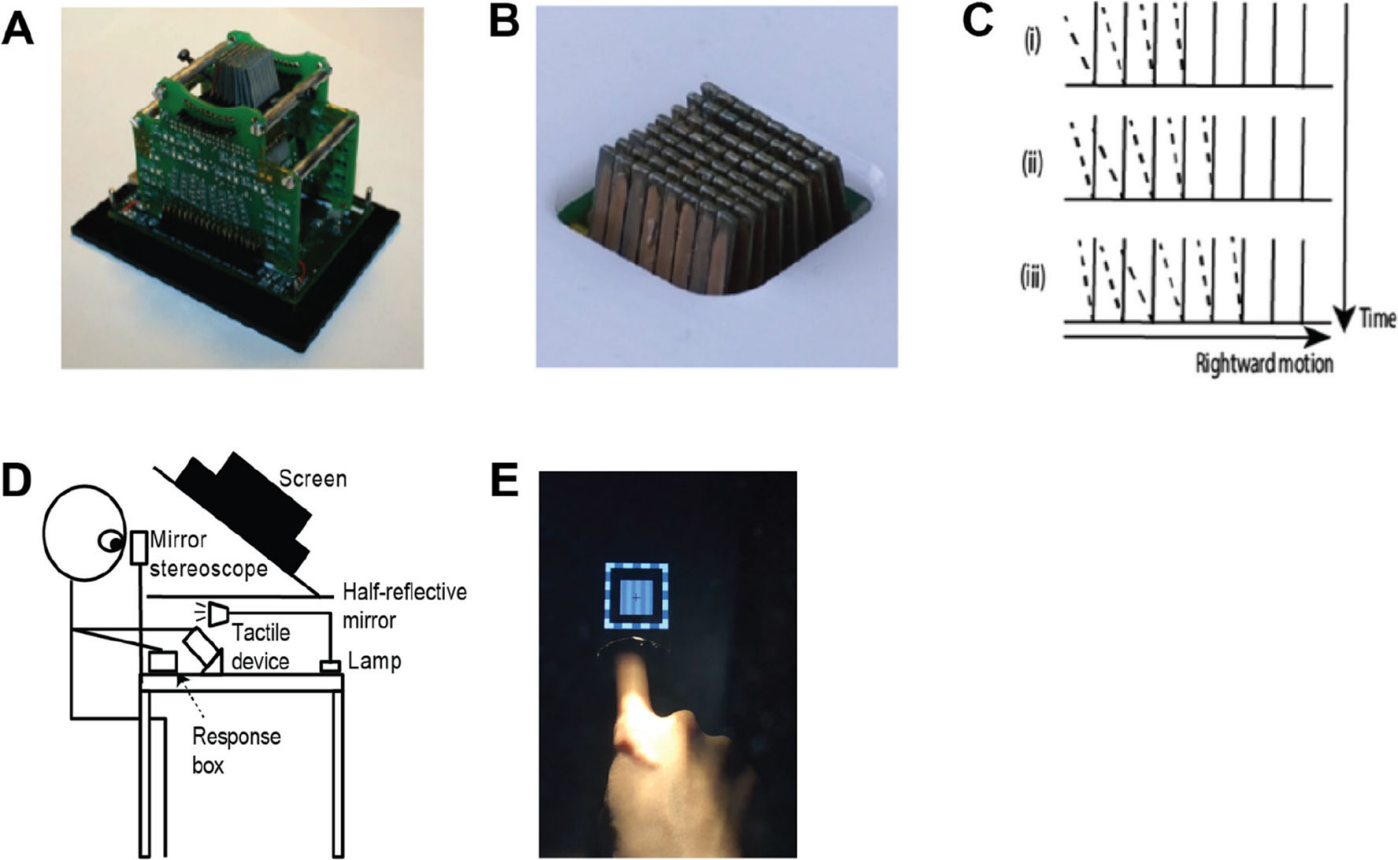
Image of the tactile device used in the experiment. **a** Close-up image of the tactile device. **b** Close-up image of the 8 x 8 pin array. **c** Side view illustration of a rightward tactile sweep across pin columns over time in consecutive frames beginning from (i). **d** Side view of the setup. Participants viewed the dichoptically presented stimuli through a mirror stereoscope and their hands were placed below a semi-silvered mirror such that there was visual feedback when the lamp was switched on. **e** An example of what the participant saw during a trial when visual feedback was available

Visual stimuli were created in MATLAB version 2017b (The MathWorks Inc., Natick, MA) using Psychtoolbox 3.0.14 (Brainard, 1997; Pelli, 1997). The stimuli were displayed on a ViewPixx custom LCD monitor (VPixx Technologies, Saint-Bruno, Canada) at a resolution of 1920 x 1080 pixels and a refresh rate of 120 Hz, and were viewed through a mirror stereoscope from a distance of 45 cm. The monitor was gamma-corrected to ensure linear luminance output and was controlled by a Mac Pro computer. The monitor was mounted on top of a wooden frame (50 x 40 x 57 cm) with the screen tilting downwards at 45^o^, and participants’ heads were supported using a forehead rest. A half-reflective mirror (41.5 x 41.5 cm) was placed approximately halfway between the monitor and the tactile device in the horizontal plane, such that the visual image reflected from the monitor appeared to be in the same spatial location and at the same distance as the haptic device placed below the mirror in conditions where visuo-tactile stimuli appeared to be spatially aligned (see Fig. 1d). A table lamp was placed behind the wooden frame so that participants’ right index or middle finger would be visible when placed on the tactile device when the light was switched on (i.e., to evaluate the effect of hand visibility; see Fig. 1e). Participants made their responses by pressing corresponding buttons (left button for the leftwards/upwards directions; right button for the rightwards/downwards directions) on a ResponsePixx button box (VPixx) with their left hand.

Visual stimuli were achromatic sine-wave gratings (visual angle 2.5^o^, spatial frequency 1.4 cpd and temporal frequency 3 Hz, mean luminance at 48 cd/m^2^, 15% Michelson contrast) presented within a square aperture on a black background. Each grating was surrounded by a square checkered frame and presented with a centered fixation cross to stabilise binocular fusion. Visual gratings either drifted along the horizontal (leftward/rightward motion) or vertical (upward/downward motion) axis.

The tactile device was a Latero Controller (Tactile Labs, Inc, Québec, Canada) consisting of 8 x 8 independent, laterally moving pins that could be deflected leftward or rightward. Each pin is controlled by a piezoelectric actuator forming an array of 64 laterally-moving skin contactors within a total area of 1.2 cm^2^. The tip of each pin could be deflected towards the left or right by approximately 0.1 mm in a triangular envelope, deforming the skin of the fingerpad resting on the device. For example, as illustrated in Fig. 1c), the leftward-most pin column would first deflect to the left before returning to the original upright position at half the deflection speed, thereby resulting in a rightward-perceived movement. Subsequent pins along each row would deflect consecutively until the rightward-most pin returned to the upright position, completing a unidirectional tactile sweep. This movement across pin columns essentially created the perception of tactile motion by skin stretch across the fingerpad. Each tactile sweep could either propagate in the leftward or rightward direction across vertical pin columns and were delivered at a rate of 1 sweep/second.

### Experimental Procedure

**Fig. 2:**
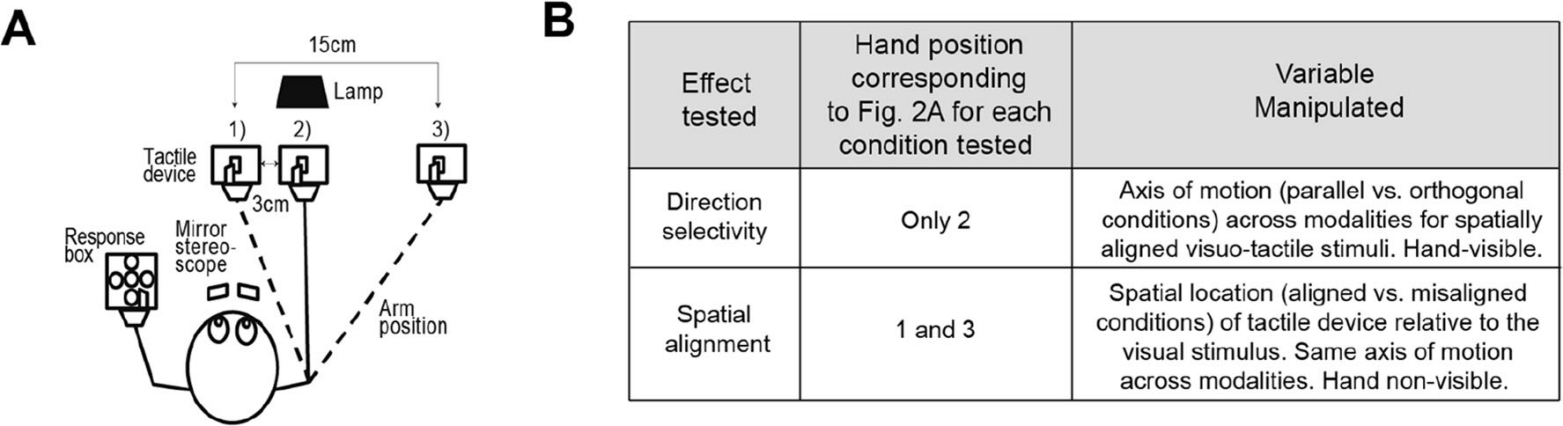
**a** Top view of the experimental setup. Participants tracked the perceived direction of the visual grating by pressing corresponding response buttons using their left hand, while their right index or middle finger (alternated over trials) was placed on the metal pins of the tactile device. Position 1 corresponds to when the tactile device was placed along the midline of the body for the spatially aligned condition when testing for the effect of spatial alignment, and when visual feedback was unavailable. Position 2 (3 cm from Position 1) corresponds to the position of the tactile device when testing for the effect of direction selectivity when visual feedback was available. Position 3 corresponds to the location of the tactile device when it was spatially misaligned with the visual stimulus (15 cm to the right of the body midline), and when visual feedback was unavailable. **b** Table summarising the variable manipulated for each effect tested (i.e., direction selectivity and spatial alignment) as illustrated in A). The effect of hand visibility was tested post-hoc by comparing the results obtained from Position 2 trials when testing for the effect of direction selectivity (Hand-visible; horizontal motion across modalities), and Position 1 trials when testing for the effect of spatial alignment (Hand non-visible; horizontal motion across modalities).

Each participant completed a total of two sessions for this experiment. One session tested whether direction selectivity across visuo-tactile stimuli influenced rivalry dynamics. In the other session, the spatial alignment of visuo-tactile stimuli was manipulated to test its effect on visuo-tactile interactions during rivalry. Each session was conducted on separate days (4 days apart on average) to prevent any adaptation artefacts, and the order in which participants were tested for each effect was counterbalanced. Each session comprised thirty-two trials split into four main blocks, and lasted approximately an hour. The effect of hand visibility on multisensory integration during rivalry was examined post-hoc by extracting and comparing the data across sessions for when the visual and tactile stimuli shared a parallel axis of motion and were spatially aligned. The variable manipulated in each session is described in Fig. 2b.

To test for the effect of direction selectivity, rivalry dynamics were compared between trials where visual and tactile stimuli moved along parallel versus orthogonal axes. As tactile sweeps presented were always laterally-moving (either rightward or leftward), a parallel axis of motion corresponded to when visual and tactile stimuli moved along the same horizontal axis of motion (i.e., rightward and leftward motion presented to each eye). To create orthogonal axes of motion across modalities, visual gratings moved along a vertical axis instead (i.e., upward and downward motion presented to each eye). The direction of visual motion rivalry (vertically or horizontally drifting gratings) was randomised and interleaved across trials. To test for the effect of spatial alignment, all trials involved the visual grating drifting parallel to the tactile stimulus, and the tactile device was either aligned with the visual stimulus along the body’s midline or was displaced 15 cm to the right from the original centered position. The tactile device was shifted 3 cm to the right of the midline for the hand-visible condition (i.e., when testing for the effect of direction selectivity) as compared to the hand non-visible condition (i.e, when testing for the effect of spatial alignment) so that the hand would be visible through the mirror stereoscope in the periphery of the right eye’s image when assessing the effect of hand visibility. Specifically, participants were able to see the length of their right index or middle finger throughout (see Fig. 1e) but not the fingertip resting on the tactile device so as to keep the luminance of the visual stimuli constant across the left and right eyes since the additional visual feedback was only visible to the right eye. Trials were blocked and counterbalanced across participants when testing for spatial alignment (i.e., spatially aligned vs. spatially misaligned blocks) effects.

During each session, prior to the main experimental blocks, participants were first required to complete a visual calibration task to align the dichoptically presented visual stimuli for the left and right eyes. During calibration for the hand-visible blocks, the position of the visual stimulus was adjusted to approximately overlap with the fingerpad that was placed on the tactile device so that visual and tactile stimuli still appeared to be spatially aligned (See Fig. 1e). Participants also had to complete a short tactile direction discrimination control task of one block consisting of 10 trials to ensure that they were able to accurately discern the direction of the tactile sweeps. Participants were asked to respond as fast and as accurately as possible (within a response window of 1 s) using button presses with their left hand immediately after each tactile sweep to indicate the direction perceived. Performance was similar across both sessions and participants generally had no difficulties in determining the direction, with accuracies within the range of 80% to 100% (M = 89.9%). Subjects were also familiarised with the task by completing a visual-only practice block that consisted of 16 trials in total: 8 binocular rivalry trials (40 s each) and 8 short catch trials to ensure that participants understood the task and were not responding randomly (both eyes were presented with the same image for 5 s each). No tactile stimulation was presented during the visual practice block. Participants were instructed to continuously track their perceptual alternations by pressing and holding the button that corresponded to the direction of visual motion perceived (i.e., the dominant percept). They were also asked to release both buttons whenever they perceived a mixture percept.

**Fig. 3:**
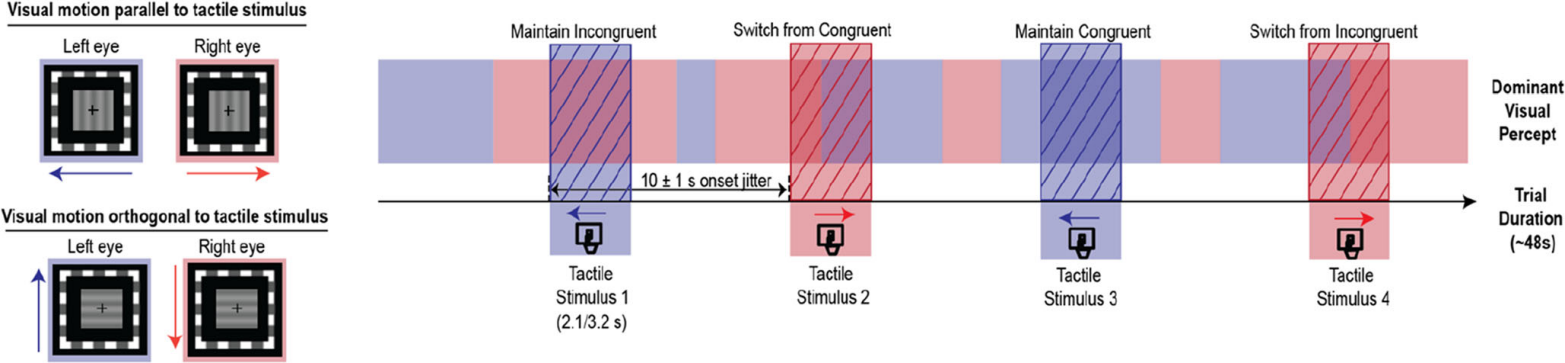
Example of alternating visual percepts experienced by a participant in one trial. Each trial consisted of four tactile stimulations (represented by segments with diagonal lines; 2.1 s for fast-switchers and 3.2 for slow-switchers) that occurred every 10 s with a 1 s timing jitter and lasted for 48 s. Correspondingly, each trial was divided into four segments from tactile onset. Visual stimuli were gratings that drifted in opposite directions for each eye. Depending on the condition, visual stimuli were oriented either parallel or orthogonal to tactile motion (leftward/upward-drifting tactile stimuli and visual percepts indicated in purple and downward/rightward-drifting in pink for illustration purposes). Four possible types of visual percepts are illustrated in terms of visuo-tactile congruency during rivalry. The congruency of each segment was defined by comparing the direction of the dominant visual percept with that of tactile motion at the beginning of tactile stimulation. Matching colour blocks during tactile stimulation therefore represent visual percepts congruent with tactile motion direction while non-matching coloured blocks represent incongruent visual percepts. If a perceptual switch occurred during tactile stimulation, the segment was categorised as a “switched” percept, else it was categorised as a “maintained” percept

For the main trials, participants pressed a key to initiate each trial and were then presented with the drifting gratings. Within each trial, two gratings, drifting in opposite vertical or horizontal directions depending on the condition, were presented. Regardless of condition, the grating directions were counterbalanced between eyes (outwards versus inwards or upwards versus downwards) across trials. Sine-wave phases for gratings were also randomised to start at different phases for each eye on every trial to prevent any adaptation effects. Each trial lasted approximately 48 s on average, and consisted of 4 tactile motion stimuli, with the directions of the tactile sweeps randomised and counterbalanced within each trial. The first tactile stimulus of each trial occurred 8 s from trial onset after binocular rivalry had stabilised. Each subsequent tactile stimulus occurred every 10 s on average, with an onset jitter of 1 s (see Fig. 3).

Participants were required to alternate between their right index and middle fingers on the tactile device across trials for the practice and main blocks to ameliorate any habituation effects. The duration of the tactile motion stimulus was calibrated according to the mean dominance durations of each participant (as obtained from the practice block). Dominance durations were defined as the time intervals participants reported viewing a visual grating to be drifting towards in one direction. Shorter dominance durations on average would indicate a faster rate of alternation between two percepts and vice versa for longer dominance durations. Considering individual differences in mean dominance durations during rivalry (Brascamp et al., 2018), the number of sweeps in a single tactile stimulus for each individual was adjusted to maximise the effect of tactile signals on vision. For instance, each tactile stimulus consisted of 2 sweeps if participants’ mean dominance duration was less than 3 s (5/14 participants) and 3 sweeps if their mean dominance duration was longer (9/14 participants). The number of tactile sweeps used for each tactile stimulus was kept the same across conditions for each participant. A 100 ms pause between each tactile sweep was added so that the direction of each discrete tactile sweep would be more distinctly felt. The tactile motion stimulus therefore lasted either 2.1 s (for “fast rivalry switchers”) or 3.2 s (for “slower rivalry switchers”). Tactile stimulation was intermittent during extended rivalry trials so that participants would not be able to predict tactile onset, thereby maintaining its salience (Lunghi & Morrone, 2013). Tactile stimulation was limited to a maximum of 3 sweeps to avoid habituation that might occur with longer stimulation periods.

## Analysis

### Multisensory congruency during rivalry

As each trial included four tactile stimulations, each trial was divided into four segments such that each segment covered a single tactile event. The start of each segment was defined from the beginning of tactile onset. In this paper, segments are categorised according to multisensory congruency at tactile onset. For example, if the direction of dominant visual motion at tactile onset matched that of the tactile motion direction, the segment would be labelled as ‘Congruent’. For each segment, if the direction of visual motion remained unchanged throughout tactile stimulation, it would be considered as a “maintained” percept. On the other hand, if the direction of the dominant visual percept switched to the previously suppressed percept, it would be considered as a “switched” percept. Four outcomes are thus possible, as shown in Fig. 3. If tactile stimulation interacts with rivalry dynamics by promoting multisensory congruency, the probability of maintaining the same visual percept during tactile stimulation would be expected to be higher for ‘Congruent’ segments. Likewise, the probability of switching for ‘Incongruent’ segments would be higher, as the suppressed ‘Congruent’ visual percept is brought back to awareness (e.g., Lunghi & Morrone, 2013). Essentially, tactile stimulation would help to resolve any visual ambiguities by maintaining congruence or by prompting a perceptual switch to maintain it.

### Statistical reports

Analyses were performed using MATLAB version 2015b (The MathWorks Inc., Natick, MA) and RStudio version 1.0.136 (R Foundation for Statistical Computing) running R version 3.2.4. ​​

### Data preprocessing

Dominance durations that were less than or equal to 180 ms were considered to be artefacts and were discarded. Time periods with no button presses indicating mixed percepts and periods in which both buttons were pressed simultaneously were also removed. For analysis purposes, the upward and downward responses for when the visual grating drifted orthogonally to the tactile stimulus were mapped to the leftward and rightward directions respectively, similar to the buttons assigned for participant responses for the Orthogonal motion condition. This arbitrary assignment allowed for a definition of tactile-visual motion direction congruency and thus for a comparison across conditions (i.e., direction of visual motion perceived).

### Generalised linear mixed-effect models to assess the probabilities of switching or maintaining percepts during tactile stimulation

**Fig. 4:**
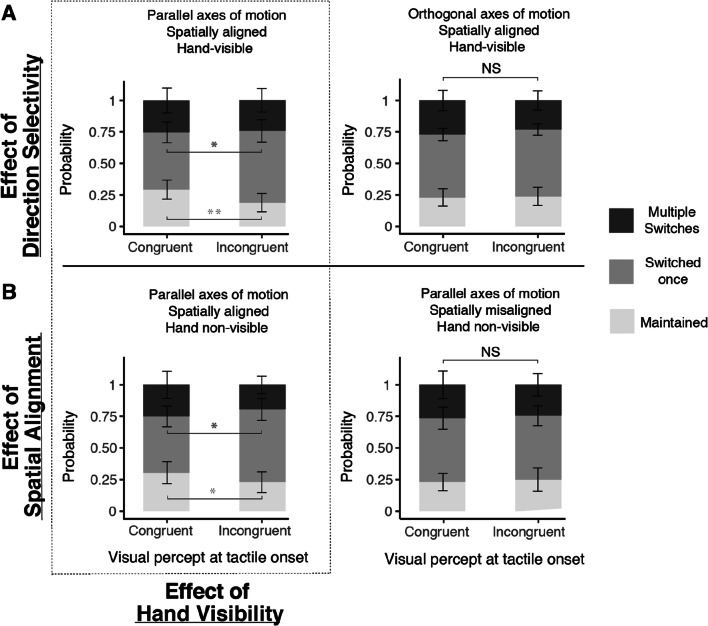
The mean probabilities of maintaining a dominant rivalry percept (light-grey bars), switching visual perception once (mid-grey bars), or switching more than once (dark-grey bars) during periods of tactile stimulation. A visual percept was classed as congruent if its motion direction matched that of the tactile stimulus at tactile onset and vice versa. Tactile stimulation significantly increased the probability of maintaining the same visual percept when tactile motion was congruent with the perceived visual stimulus provided the visual and tactile motions were parallel and aligned, regardless of whether the hand was visible or not (cf. Panels A (left) and B (left). Asterisks indicate results from paired, two-tailed *t*-tests: * = *p* ≤ .05, ** = *p* ≤ .01). The probability of switching to the suppressed visual percept was also significantly higher when the tactile motion was incongruent with the perceived visual stimulus. On the other hand, Panels A (right) and B (right) show that tactile stimulation had no effect on binocular rivalry dynamics (no significant [NS] differences across all three response types) when the visual and tactile stimuli moved in orthogonal directions (A; Right) or when the stimuli were parallel but spatially displaced (B; Right). Error bars represent group 95% confidence intervals for each response type (refer to Table 1 in Supplementary section for specific probability values illustrated in Fig. 4)

To quantify the effects of tactile motion on rivalry dynamics under each condition, the probabilities of maintaining or switching visual percepts during tactile stimulation were computed (Fig. 4). Tactile effects were assessed using generalised linear mixed-effects models on segment-wise data across participants. The *glmer()* function from the *lme4* package was used to compute parameter estimates (Bates et al., 2015) and the *mixed()* function from the *afex* package in R was used to obtain *p*-values for the terms in each model using likelihood ratio tests (Singmann et al., 2021; Singmann & Kellen, 2019). Three separate full models were run, one for each effect of interest (i.e., direction selectivity, spatial alignment and hand visibility). Segments containing mixed percepts or multiple switches were excluded for this analysis. The dependent variable of the model corresponded to the number of switches within each segment: 1 if a perceptual switch occurred and 0 if no switches occurred (i.e., a maintained percept). Within each model, fixed covariates included the effect of interest (categorical with two levels e.g., parallel vs. orthogonal axes of motion as within-factor levels for the effect of direction selectivity) and the initial congruency of visual percept relative to tactile motion at tactile onset (congruent vs. incongruent as within-factor levels). Of particular relevance to the aim of this study was that each binomial model included an interaction between the two terms so as to examine the influence of bimodal manipulation on rivalry dynamics. Each model also included random intercepts to account for individual biases and was fit with the logit link function. Each full model was compared against a reduced version of itself to compare the goodness of fit of the two models based on the log-likelihood ratio. The reduced model included the same fixed covariates and random intercepts as the full model, except without an interaction between the fixed covariates. Effect size estimates, standard errors, *z*- and *p*-values obtained for the terms in each model are included in the Supplementary section.

Where significant two-way interactions were observed, follow-up pairwise contrasts were carried out on the full model using the *pairs()* function in the emmeans package (Lenth, 2018): the estimated marginal means on the response scale (i.e., measured in terms of probability after the application of the inverse link function) for each level of a factor (e.g., congruent vs. incongruent for initial congruency) were contrasted separately with each level of the other factor (e.g., Parallel vs. Orthogonal motion trials).

### Segment extraction for binocular rivalry timecourse data to examine tactile influences on visual percepts

**Fig. 5:**
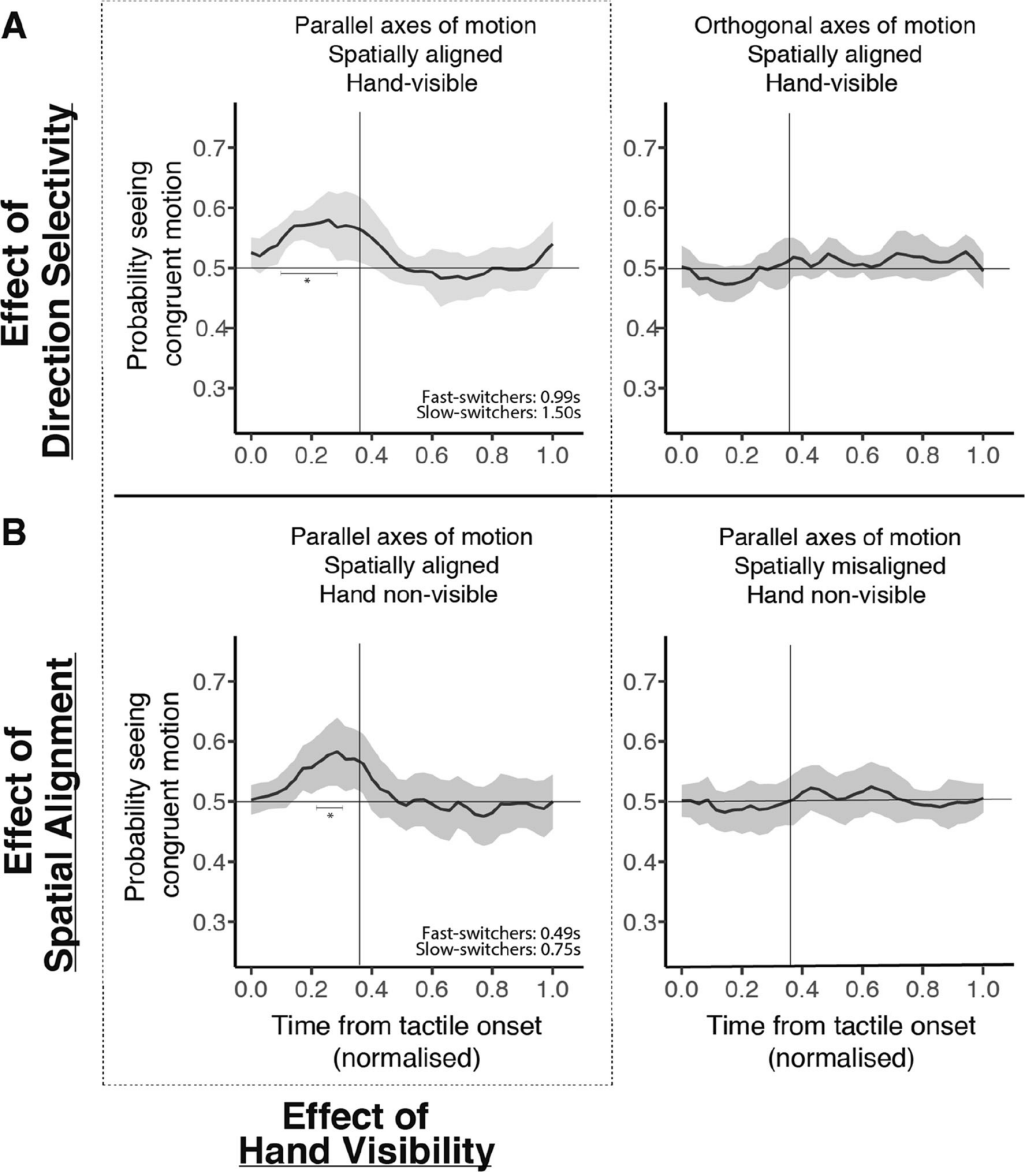
Group mean data (thick black line) showing the probability of the dominant visual motion being congruent with the direction of tactile motion across normalised time since tactile onset. The shaded area shows the 95% CI around the mean for each bin. The durations included in the left panels of A and B indicate the length of time that the waveform was significantly above chance (FDR-corrected; horizontal line corresponds to chance level at 0.5) for the fast and slow switch groups. These timings were obtained by multiplying the number of significant bins within the extracted time segment with the actual segment length extracted for each switch group. Crucially, only when visual and tactile motion shared a parallel axis of motion, and were spatially aligned did the timecourse show significant elevations above chance within the period of tactile motion (the vertical line shows the normalised tactile motion duration). In contrast, when the visual and tactile stimuli were orthogonal to each other or were in different locations, the probability trace remained flat and never differed significantly from chance (*t*-tests, two tailed, α = .025)

**Fig. 6:**
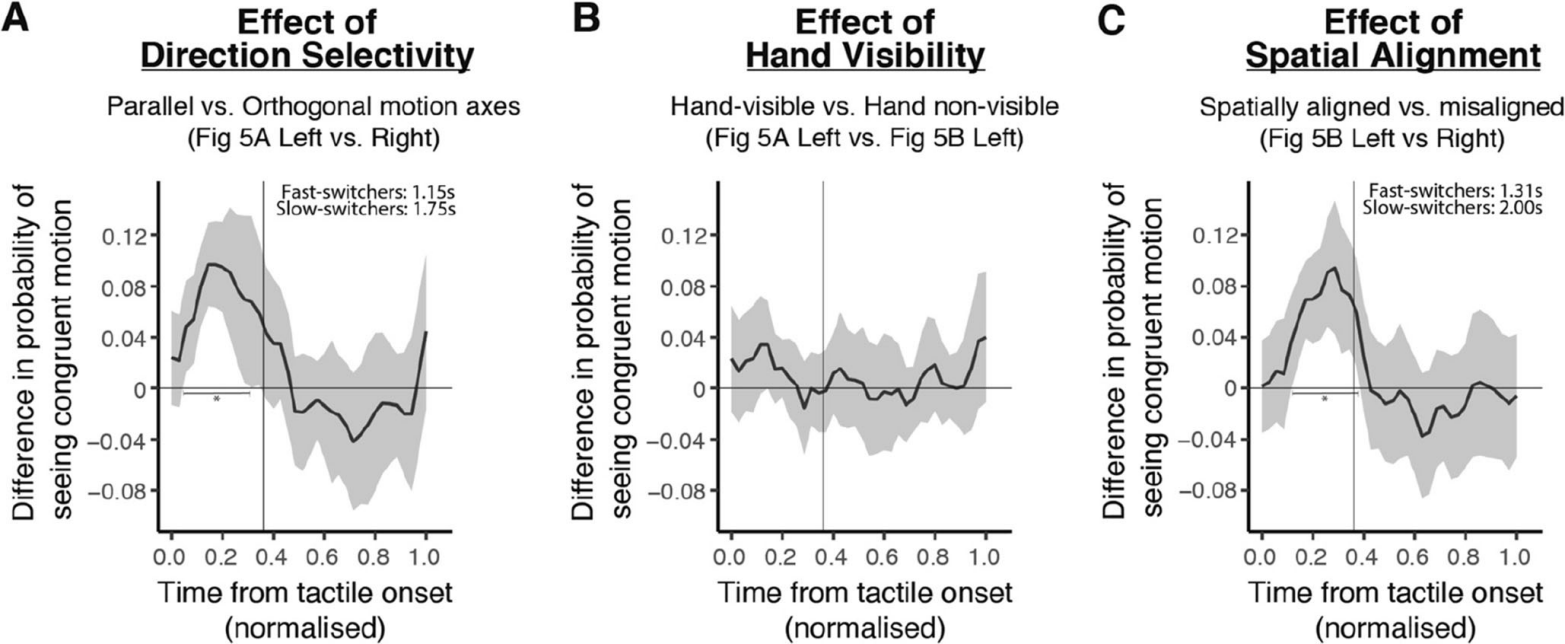
Group mean data (thick black line) showing the effect of direction selectivity (Panel A), hand visibility (Panel B) and spatial alignment (Panel C) defined as the difference between the waveforms as shown in Fig. 5 and plotted as a function of normalised time from tactile onset. The shaded area shows the 95% CI around the mean data. Only panels A and C show significant differences across conditions during tactile stimulation (horizontal line corresponds to no difference; vertical line shows normalised tactile motion duration). In comparison, the effect of hand visibility did not reach statistical significance. The durations included in Panels A and C indicate the length of time that the waveforms significantly differed from chance for the fast and slow switch groups, and correspond to the number of significant bins in terms of the original segment lengths extracted

We next looked at the effect of tactile stimulation on the dynamics of binocular rivalry as a timecourse measure. The instantaneous probability of the dominant visual percept being congruent with the direction of the tactile stimulus from tactile onset was computed for each condition (Fig. 5). Taking into account the different tactile stimulation durations across the slow- and fast-switch groups, different segment lengths were extracted for each group so that tactile stimulation as a proportion of segment length would be the same when aggregating data across participants (as illustrated in Figs. 5 and 6). Segments were cut-off 9 s from the beginning of tactile onset for the slow-switchers, as this was the maximal duration before the next tactile onset (9 participants; mean dominance duration across conditions: 3.53 s ± 0.17 [M ± SD]) while segments were cut-off at 5.91 s for the fast-switchers (5 participants; mean dominance duration across conditions: 2.16 s ± 0.25). Each segment was normalised between a range of 0 to 1, so that tactile stimulation took up 0.36 of each segment (corresponds to vertical lines in Figs. 5 and 6: 3.2/9 s [slow-switchers] or 2.1/5.91 s [fast-switchers]). Timecourses within each segment were binned into 36 bins. Data were first averaged across segments to obtain an overall timecourse for each participant per condition then averaged across participants to obtain Fig. 5. Tactile onsets were aligned at time zero across segments regardless of their visuo-tactile congruency at tactile onset, so that each probability trace started around chance level (0.5). Each bin was compared to chance level using a one-sample *t*-test (two-tailed, ɑ = .025) to evaluate the effect of tactile stimulation on rivalry dynamics for each condition (Lunghi & Morrone, 2013). To control for multiple comparisons, *p*-values across bins for each timecourse were corrected using false discovery rate (FDR; Benjamini & Hochberg, 1995).

To evaluate the extent of influence the effects of direction selectivity, spatial alignment, and hand visibility had on visuo-tactile integration dynamically, we computed the difference between the waveforms of the respective conditions used to assess each effect across participants (Fig. 6). For instance, the waveform for when orthogonal axes of motion was presented across modalities was subtracted from the waveform for when a parallel axis of motion was presented to evaluate the significance of the alignment of motion axes on visuo-tactile interactions. Cluster permutation tests were applied on the timecourse data to correct for multiple comparisons (refer to Figure 1 in Supplementary section for results of cluster permutation test). ​​For each permutation, responses for each participant were randomly shuffled across bins and conditions, and the difference between conditions over time was obtained across trials. This difference was transformed into a *t*-statistic for each bin and *t*-values were compared to a specified threshold, which corresponds to the 97.5^th^ quantile of a *T*-distribution for two-sided *t*-tests, at an alpha-level of 0.025. Samples that were larger than the specified threshold were clustered in terms of temporal adjacency based on the positive or negative cluster values and the sum of *t*-values within a cluster was computed. ​​The largest cluster size in absolute value was extracted for each permutation. This process was repeated 1,000 times, and the 95% permuted cluster size served as a significance limit that was compared against the actual observed cluster mass (Maris & Oostenveld, 2007).

### Segment extraction to examine the time taken for the first perceptual switch to occur from tactile onset

**Fig. 7:**
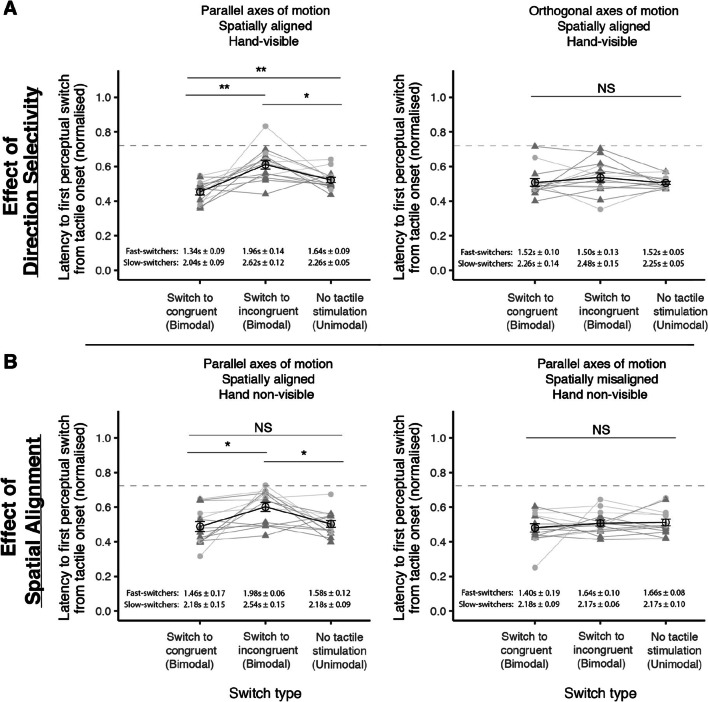
Time taken for the first perceptual switch to occur from tactile onset relative to a visual-only baseline. Two kinds of switches were possible when tactile stimulation was present: (1) a switch to congruence (perceived visual direction conflicts with the tactile direction at tactile onset), or (2) a switch to incongruence (perceived visual direction matches that of tactile direction at tactile onset). Each data point represents a single participant (dark grey, filled triangles: slow-switchers; light grey, filled circles: fast-switchers). The horizontal grey dotted line indicates touch duration (0.75 of each normalised time segment), and the black points indicate the mean switch times across all participants. When visual and tactile stimuli shared a common axis and were spatially aligned, switches to congruence occurred significantly earlier in the touch period than switches to incongruence, and switches to incongruence occurred significantly later than the visual-only baseline regardless of hand visibility (Panels A and B; Left). However, switches to congruence only occurred significantly earlier than baseline when the hand was visible. No differences in switch times across segment types were observed when the motion of axis and spatial location across visuo-tactile stimuli were not aligned (Panels A and B; Right). The durations included in each panel correspond to the mean timings for each switch group in terms of seconds. Results from paired-sample *t*-tests are shown by asterisks: * = *p* < .05; ** = *p* < .01. Error bars represent the standard error of mean (±1 SEM) for each condition

To further assess the influence of tactile stimulation on rivalry dynamics, the time taken for participants to experience the first perceptual switch in the presence and absence of tactile motion for each effect was also examined. Specifically, the time taken for a perceptual switch to the direction that is congruent or incongruent with the tactile stimulus for bimodal segments was compared against a neutral visual-only baseline across conditions (Fig. 7).

In order to create unimodal (i.e., visual only) and bimodal (i.e., visual and tactile) segment types and to prevent any temporal overlaps between consecutive segments (Fig. 7), segment lengths lasted for approximately half the segment durations extracted for the timecourse data (i.e., segments were 4.25 s long for the slow-switchers and 2.95 s for the fast-switchers). Tactile stimulation duration as a proportion of segment length was therefore still similar across switch groups, and unimodal and bimodal segments were of the same length. Unimodal segments preceded bimodal segments and were marked backwards from the onset of each tactile stimulus while bimodal segments were calculated from the beginning of tactile stimulation, resulting in the same number of unimodal and bimodal segments per participant. For the unimodal segments, the time taken for the first perceptual switch to occur was calculated from the beginning of each segment and subsequently averaged across segments regardless of the visual direction perceived. Only segments including one perceptual switch were included in this analysis regardless of segment type.

To allow for the aggregation of data across switch groups​​, the time taken for a perceptual switch to occur was first converted as a proportion of segment length. The mean time taken to switch for each segment type was then obtained for each participant and subsequently averaged across participants. For analysis of variance (ANOVA) tests, Greenhouse-Geisser corrections were applied to *F* and *p*-values whenever Mauchley’s sphericity test indicated a significant violation. Bonferroni correction was used, with original *p*-values multiplied by the number of segment types within each condition.

## Results

### Probabilities of switching or maintaining percepts for ‘Congruent’ and ‘Incongruent’ segments across conditions

Results from likelihood ratio tests comparing the full and reduced mixed logistic regression models for each effect showed that the full model provided a better fit, with significant two-way interactions between the factors of initial visuo-tactile congruency and condition observed for the effects of direction selectivity (χ^2^ (1)= 7.64, *p* = .006), and spatial alignment (χ^2^ (1) = 5.77, *p* = .016) but not for hand visibility (χ^2^ (1) = 0.43, *p* = .511). Follow-up pairwise contrasts for each model ran indicated a significant increase in the probability of visual percepts switching to match that of the tactile stimulus only when both visual and tactile stimuli shared a common axis of motion and when they appeared to be spatially aligned (Parallel motion axes condition for the effect of direction selectivity [Fig. 4a; Left]: *z* = -3.67, *p* < .001; Spatially aligned condition for the effect of spatial alignment [Fig. 4b; Left]: *z* = -2.87, *p* = .004). In line with expectations, these results suggest that the tactile stimulus was able to curtail suppression periods for direction-congruent visual stimuli when the motion axes and spatial location across both modalities coincided regardless of hand visibility. On the other hand, the direction of the tactile stimulus failed to bias the probability of switching to either direction when orthogonal axes of motion were presented across modalities (Fig. 4a; Right: *z* = 0.17, *p* = .864), and when visuo-tactile stimuli were spatially misaligned (Fig. 4b; Right: *z* = 0.57, *p* = .570)

### Binocular rivalry timecourse dynamics

Across the conditions where the visuo-tactile stimuli shared a parallel axis of motion and were spatially aligned (left panels of Fig. 5a and b), the probability of seeing a congruent visual percept only increased after tactile stimulation and decreased back to chance level upon tactile offset, suggesting that the switch to congruency and the subsequent maintenance of the visual percept was closely tied to the tactile stimulus. The size of the effect in Fig. 5b (Left) as measured by the peak probability of the congruent percept in this study is also comparable to the results obtained for the passive touch condition in Lunghi and Morrone (2013). In their study, the passive touch condition included a spatially collocated tactile grating that drifted back and forth on participants’ fingerpads similar to the spatially aligned, hand non-visible condition in this study, which might have induced perception of motion, albeit to a minimal extent. On the other hand, when the visual and tactile stimuli were not spatially overlapped and/or did not match in direction, the average probability trace remained flat and was not statistically different from chance level throughout, indicating that sensory signals across modalities failed to combine.

The finding that tactile sweeps only had significant effects on visual percepts when the axes of motion and spatial location across visuo-tactile stimuli were aligned is further illustrated in Fig. 6. This demonstrates a sensory congruency effect, highlighting these criteria as mandatory for visual and tactile integration as evident from studies that have found enhancement and inhibitory effects across modalities when these conditions were fulfilled (Gori et al., 2011; Ide & Hidaka, 2013). On the contrary, hand visibility during tactile stimulation did not yield a significant enhancement in general, and does not appear necessary for multisensory integration.

### Time taken for the first perceptual switch to occur from tactile onset

Given that no significant effects of hand visibility was observed overall, this analysis only included the effects of direction selectivity and spatial alignment. Separate 2 x 3 repeated-measures ANOVAs were run for each effect with the relevant Condition (i.e., parallel vs. orthogonal axes of motion for direction selectivity) and Segment type (bimodal congruent and incongruent segments, and unimodal segments) as within-subject factors.

A significant main effect of segment type (*F*(1.93,25.03)=12.99, *p* < .001, η_G_^2^ = .22) was accompanied by a significant interaction between the two factors (Condition x Segment type; *F*(1.50,19.48)=5.05, *p* = .024, η_G_^2^ = .12) for the effect of direction selectivity. The significant interaction was likely due to the differences in timings observed when visuo-tactile stimuli shared a parallel axis of motion. Follow-up pairwise comparisons showed that the time taken for the first perceptual switch to occur across all three segment types differed significantly from each other, with a delay in switching to the incongruent direction (0.61 ± 0.03 [M ± SD]) as compared to switches to the congruent direction (0.45 ± 0.02; *t*(13)=4.48, *p* = .002) and when tactile stimulation was absent (0.52 ± 0.02; *t*(13)=2.90, *p* = .037). Importantly, switches to the congruent direction occurred earlier than the visual-only baseline (*t*(13)=-3.51, *p* = .011), consistent with previous results illustrating that the temporal dynamics of binocular rivalry can be modulated with tactile stimuli even in the absence of awareness by bringing the suppressed percept back to dominance based on spatial, temporal and direction congruency (Lunghi & Alais, 2015; Lunghi & Morrone, 2013). In contrast, the time taken to switch to either direction for the bimodal segments did not significantly differ from each other and from the unimodal baseline when stimuli moved along orthogonal axes (Panel A, Right; Incongruent vs. Congruent: *t*(13)=1.02, *p* = .327; Congruent vs. Baseline: *t*(13)=0.10, *p* = .925; Incongruent vs. Baseline: *t*(13)=1.37, *p* = .194).

A significant main effect of segment type (*F*(2,26)=3.50, *p* = .045, η_G_^2^ = .11) and interaction was also observed for the effect of spatial alignment (*F*(1.89,24.51)=5.10, *p* = .015, η_G_^2^ = .07). Similar to the trends observed in the Parallel motion condition for the effect of direction selectivity, incongruent switches (0.60 ± 0.03) occurred significantly later than switches to the congruent direction (0.49 ± 0.03; *t*(13)=3.15, *p* = .023) and in the absence of tactile stimulation (0.50 ± 0.02; *t*(13)=2.97, *p* = .033) when visuo-tactile stimuli were spatially aligned. Switches to the congruent direction, however, did not occur significantly earlier as compared to the unimodal baseline (*t*(13)=-0.39, *p* = .703). Taken together, the results suggest that congruent tactile stimulation mainly stabilised rivalry dynamics by promoting dominance of an already congruent visual percept. The results obtained for when the visuo-tactile stimuli were spatially misaligned mirrored that for the Orthogonal motion condition: the presence and/or absence of tactile stimulation did not affect visual percepts (Panel B, Right; Incongruent vs. Congruent: *t*(13)=0.83, *p* = .421; Congruent vs. Baseline: *t*(13)=-0.99, *p* = .339; Incongruent vs. Baseline: *t*(13)=-0.22, *p* = .828).

## Discussion

In the current study, we used rivalry perceptual fluctuations to investigate interactions between visual and tactile motion signals. Using tactile motion sweeps delivered to the right index or middle fingerpad and translating visual gratings, we tested the extent to which the alignment of motion axes and spatial alignment of the visual and tactile stimuli plays a role in visuo-tactile integration. We found that visual and tactile motion share a direction-specific component and that spatial proximity was necessary for cross-modal integration to occur, in accord with previous studies (Gepshtein et al., 2005; Lunghi & Morrone, 2013). In addition, we found that visibility of the hand during touch did not provide a significant advantage relative to a hand-invisible condition, similar to what James and Blake (2004) observed when participants viewed a rotating sphere enclosed in their hand. Our findings extend existing literature to show that touch can influence visual perception for dynamic tasks involving motion and also have implications for visuo-proprioceptive associations (Hense et al., 2019; Lunghi et al., 2010; Lunghi & Morrone, 2013; Maruya et al., 2007).

### Direction selectivity across vision and touch

​​In this study, it was observed that visuo-tactile interactions occurred exclusively when the axes of motion for visual and tactile signals were parallel to each other. Tactile motion could bias the perceived direction of rivalling visual motions by stabilising rivalry if the tactile motion was congruent with the dominant visual motion (extending dominance durations). Tactile motion could also modulate rivalry dynamics by promoting a perceptual switch to the other visual stimulus if the tactile motion was incongruent (truncating current dominance to make the suppressed image visible), although this effect was attenuated when participants were not able to view their hands. Both effects are examples of cross-modal interactions driven by congruence. The results observed makes MT a highly plausible neural substrate underlying visuo-tactile interactions, as MT has been found to encode for vertical and horizontal motion, as well as for the accurate discrimination of motion direction within the same cardinal axis (Schneider et al., 2019; van Kemenade et al., 2014). Incongruent motion across modalities might therefore activate different axis of motion clusters within MT, resulting in no sensory interaction (Zimmermann et al., 2011). In addition, neural evidence in the form of stimulus-driven and sustained gamma-band activity (GBA) in visual and somatosensory cortices have also been found to be larger for parallel compared to orthogonally moving stimuli (Krebber et al., 2015). Importantly, GBA is associated primarily with bottom-up processing, which suggests that changes in neural oscillations pertain to stimulus changes, and supports the notion that vision and touch do share underlying neural resources. Direction congruence is thus crucial in integrating visuo-tactile stimuli across sensory cortices.

### Spatial alignment between vision and touch for dynamic signals

In accord with the unity assumption, which posits that perceptual cues across modalities would be more likely to be integrated if they are perceived to originate from a common location, visuo-tactile research examining discrimination of orientation, temporal modulation and motion direction have mostly used aligned conditions (Hense et al., 2019; Lunghi et al., 2010, 2017; Lunghi & Morrone, 2013) . In this study, we found that touch failed to influence vision when the tactile device was placed 15 cm away from the visual stimulus, consistent with the spatial rule of multisensory integration shown in numerous studies, as embodied by Bayesian inference models (Körding et al., 2007; Sato et al., 2007). It also agrees with neuroimaging evidence showing that visual extrastriate areas and somatosensory parietal operculum activity are modulated by spatial congruence, with stronger activations observed when visual and tactile stimuli are delivered to the same contralateral location concurrently. Furthermore, these sensory-specific areas showed cross-modal spatial effects regardless of modality and task-relevance, showing that spatial alignment is mandatory for combining visual and tactile signals (Macaluso et al., 2002, 2005).

In our study, the tactile device was not directly aligned with the body’s midline but was offset slightly to the right for the hand-visible conditions. Interestingly, the results show that as long as the binocularly fused visual image appears to be spatially aligned with the tactile stimulus, tactile motion can still influence visual percepts. This underscores the functional association of spatial representations across vision and touch, such as visual-somatosensory maps coded in eye-centered coordinates and updated by gaze position, which highlights the fact that vision is able to localise touch (Macaluso et al., 2002). As seen from Fig. 5, switches to the congruent and subsequent switches back to incongruent directions were tied to tactile onset and offset, respectively. This suggests that cross-modal interactions might still occur when the visual stimulus is sufficiently close to the location of the hand in space, regardless of the availability of visual feedback such as hand visibility, and when tactile sweeps correspond to the visual image in retinal space (Soto-Faraco et al., 2004). Our results are in line with previous findings of visuo-tactile spatial ventriloquism, whereby the location of the tactile stimulus in relation to our body and external space can be remapped based on visual information, and illustrates that dynamic alignment between the two sensory spatial maps can occur without the need for any active exploratory action of participants (Badde et al., 2020; Blake et al., 2004; Bruno et al., 2007; Hu & Knill, 2010; Samad & Shams, 2016).

It might be argued that the directional congruency effects observed in the present study were mediated by non-sensory factors, such as participant response bias. Although participants were asked to press the relevant button corresponding to the visual direction currently dominant, and to release any button when perceiving a mixed percept, participants might have been biased to respond to the direction of the tactile stimulus, regardless of their visual percept. Even if this happened only transiently at the onset of the tactile motion, it would tend to produce similar data patterns to what we report here for switching visual percepts or maintaining them. However, the pattern of results observed in this study are similar to that reported by Hense et al. (2019), where visual and tactile stimuli moved along a vertical axis instead. These results suggest that tactile motion can interfere with rivalry dynamics as long as both modalities share a common axis of motion, possibly arguing against a purely response bias account.

### Hand visibility: The presence of visual contextual feedback and multisensory interactions

Although hand visibility did result in faster congruent switches relative to a unimodal baseline (as in Fig. 7) in this study, visibility of the hand did not show a significant advantage in general. This is possibly attributed to the small size of the motion driver and the fact that participants could only view their hand location through the stereoscope, which might have contributed to inaccuracies in proprioception. It remains a possibility that being able to see the hand might still be able to serve as an ecological visual stimulus, which would be worth investigating in future studies.

### Possible mechanisms underlying visuo-tactile motion perception

Our results show that spatially aligned and directionally congruent tactile motion affected both phases of binocular rivalry, increasing the durations of already congruent percepts and decreasing durations of suppressed percepts. That tactile motion incongruent with the currently perceived visual motion can cause a perceptual switch so that the suppressed visual stimulus becomes visible to achieve cross-modal congruence is in agreement with earlier findings (Hense et al., 2019; Lunghi & Alais, 2015).​​ It is proposed that tactile stimulation is able to prevent the suppressed congruent visual image from becoming deeply suppressed and cause a perceptual switch if tactile onset occurred later during the suppression phase when visual suppression has weakened. As selective attentional control of rivalry dynamics is limited, any modulation in visual perception caused by another sensory signal implies a sensory interaction at an early level outside of awareness (Meng & Tong, 2004; Paffen & Alais, 2011). This suggests that visuo-tactile integration can take place at neural sites earlier in the processing pathway than previously thought, such as the extrastriate hMT+/V5 complex that has traditionally been associated with only the visual modality, and without awareness of the visual stimulus (Gori et al., 2011; Konkle et al., 2009; Paffen & Alais, 2011).

However, this conclusion has to be taken with caution in relation to the present study, as the shortening of suppression durations relative to a unimodal baseline was not observed when participants were not able to see their hands. This result might perhaps be attributed to the nature of the tactile stimulus that was made up of brief tactile sweeps instead of sustained continuous motion generated with a rotating wheel, for example, thereby resulting in weaker tactile stimulation. On the other hand, the enhanced congruency effect observed when participants’ hands were visible might be attributed to a heightened awareness of objects in near-hand space (Reed et al., 2006). For example, placing a hand close to a visual target was found to sharpen the orientation tuning of V2 neurons via feedback mechanisms from fronto-parietal areas, and implies the presence of bimodal visuo-tactile representations of near-hand space (Perry et al., 2015).

### Conclusions

In summary, we report clear evidence of spatially localised and directionally selective visuo-tactile motion interactions. This cross-sensory modulation of vision by touch requires spatial alignment across modalities and is eliminated by a small (15 cm) displacement of the tactile stimulus away from the visual stimulus. It is also directionally selective, as tactile motions orthogonal to the rivalling visual motions exert no influence at all on vision. This implies that visuo-tactile motion interactions occur at a low level of sensory processing where neurons are local and tuned to feature dimensions.

## Supplementary Information

ESM 1 (DOCX 205 kb)
