# Supplementary material for: Direction-selective modulation of visual motion rivalry by collocated tactile motion
Source: Atten Percept Psychophys. 2022 Feb 22;84(3):899–914. doi: 10.3758/s13414-022-02453-y (PMC9001558; doi:10.3758/s13414-022-02453-y)
Supplement: Supplementary file 1 — (DOCX 205 kb) [file 13414_2022_2453_MOESM1_ESM.docx]

**Supplementary Materials**

| **Condition** | **Visuo-tactile congruence on tactile onset** | **Number of switches per segment** | **Mean Probability** | **Lower CI** | **Upper CI** |
| --- | --- | --- | --- | --- | --- |
| - Parallel axes of motion - Hand-visible - Spatially aligned | Congruent | None | 0.29 | 0.22 | 0.37 |
|  |  | One | 0.45 | 0.37 | 0.54 |
|  |  | Multiple | 0.25 | 0.15 | 0.35 |
|  | Incongruent | None | 0.19 | 0.12 | 0.26 |
|  |  | One | 0.57 | 0.48 | 0.66 |
|  |  | Multiple | 0.24 | 0.15 | 0.34 |
| - Orthogonal axes of motion - Hand-visible - Spatially aligned | Congruent | None | 0.23 | 0.16 | 0.30 |
|  |  | One | 0.50 | 0.45 | 0.55 |
|  |  | Multiple | 0.27 | 0.19 | 0.35 |
|  | Incongruent | None | 0.24 | 0.17 | 0.31 |
|  |  | One | 0.53 | 0.49 | 0.58 |
|  |  | Multiple | 0.23 | 0.15 | 0.31 |
| - Parallel axes of motion - Hand non-visible - Spatially aligned | Congruent | None | 0.30 | 0.22 | 0.39 |
|  |  | One | 0.45 | 0.36 | 0.53 |
|  |  | Multiple | 0.25 | 0.14 | 0.36 |
|  | Incongruent | None | 0.23 | 0.15 | 0.31 |
|  |  | One | 0.57 | 0.49 | 0.66 |
|  |  | Multiple | 0.20 | 0.13 | 0.27 |
| - Parallel axes of motion - Hand non-visible - Spatially misaligned | Congruent | None | 0.23 | 0.16 | 0.30 |
|  |  | One | 0.51 | 0.42 | 0.59 |
|  |  | Multiple | 0.26 | 0.15 | 0.38 |
|  | Incongruent | None | 0.25 | 0.16 | 0.34 |
|  |  | One | 0.51 | 0.43 | 0.58 |
|  |  | Multiple | 0.25 | 0.16 | 0.33 |

**Table 1.** Table of values containing the mean probabilities of maintaining a percept (no switches within a segment), switching once, or more than once during tactile stimulation across trials and participants for each condition. Values included in the table correspond to Figure 4.

Parameter estimates for generalised linear mixed-effects models:

1. Effect of direction selectivity (* indicates *p* < .05)

Full model:

| **Fixed Effects** | **Estimate** | **Std. Error** | ***z*-value** | ***p*-value** |
| --- | --- | --- | --- | --- |
| Visuo-tactile congruence on tactile onset | -0.321 | 0.127 | -2.515 | 0.012* |
| Condition | 0.151 | 0.093 | 1.626 | 0.700 |
| Interaction | -0.351 | 0.127 | -2.579 | 0.006* |

Reduced model:

| **Fixed Effects** | **Estimate** | **Std. Error** | ***z*-value** | ***p*-value** |
| --- | --- | --- | --- | --- |
| Visuo-tactile congruence on tactile onset | -0.313 | 0.127 | -2.467 | 0.014* |
| Condition | -0.036 | 0.063 | -0.566 | 0.572 |

Follow-up pairwise contrasts:

|  | **Orthogonal axes of motion** | | **Parallel axes of motion** | |
| --- | --- | --- | --- | --- |
| Visuo-tactile congruence on tactile onset | Congruent | Incongruent | Congruent | Incongruent |
| **Probability** | 0.713 | 0.707 | 0.625 | 0.766 |
| **Std. Error** | 0.037 | 0.037 | 0.041 | 0.034 |

1. Effect of spatial alignment (* indicates *p* < .05)

Full model:

| **Fixed Effects** | **Estimate** | **Std. Error** | ***z*-value** | ***p*-value** |
| --- | --- | --- | --- | --- |
| Visuo-tactile congruence on tactile onset | -0.199 | 0.125 | -1.584 | 0.114 |
| Condition | 0.0847 | 0.089 | 0.947 | 0.294 |
| Interaction | -0.301 | 0.125 | -2.402 | 0.016* |

Reduced model:

| **Fixed Effects** | **Estimate** | **Std. Error** | ***z*-value** | ***p*-value** |
| --- | --- | --- | --- | --- |
| Visuo-tactile congruence on tactile onset | -0.210 | 0.125 | -1.682 | 0.093 |
| Condition | -0.069 | 0.062 | -1.111 | 0.267 |

Follow-up pairwise contrasts:

|  | **Spatially misaligned** | | **Spatially aligned** | |
| --- | --- | --- | --- | --- |
| Visuo-tactile congruence on tactile onset | Congruent | Incongruent | Congruent | Incongruent |
| **Probability** | 0.728 | 0.707 | 0.634 | 0.741 |
| **Std. Error** | 0.048 | 0.050 | 0.055 | 0.046 |

1. Effect of hand visibility (* indicates *p* < .05)

Full model:

| **Fixed Effects** | **Estimate** | **Std. Error** | ***z*-value** | ***p*-value** |
| --- | --- | --- | --- | --- |
| Visuo-tactile congruence on tactile onset | -0.847 | 0.409 | -2.073 | 0.038* |
| Condition | -0.225 | 0.188 | -1.198 | 0.231 |
| Interaction | 0.1664 | 0.253 | 0.658 | 0.511 |

Reduced model:

| **Fixed Effects** | **Estimate** | **Std. Error** | ***z*-value** | ***p*-value** |
| --- | --- | --- | --- | --- |
| Visuo-tactile congruence on tactile onset | -0.592 | 0.126 | -4.695 | < .001* |
| Condition | -0.133 | 0.126 | -1.061 | 0.288 |


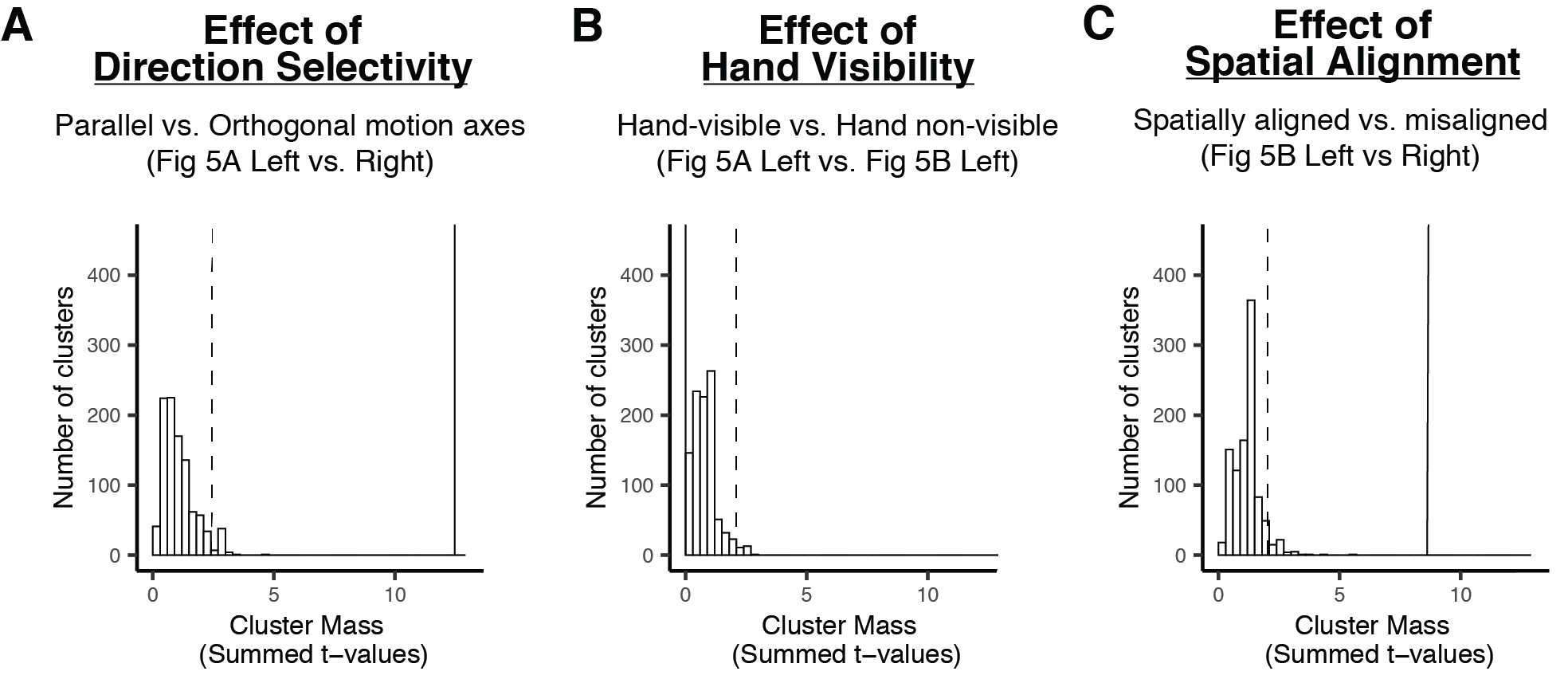


**Figure 1.** Cluster permutation test results for timecourse data of each effect tested (timecourse data corresponds to that presented in Figure 6 in the main text). The solid vertical line represents the actual cluster mass while the dashed line represents the 95% permuted cluster mass based on the largest cluster size extracted for each permutation. The actual mean cluster mass was significantly above that of the permuted cluster mass only for the effects of direction selectivity and spatial alignment (Panels A and C).
